# 3Mont: A multi-omics integrative tool for breast cancer subtype stratification

**DOI:** 10.1371/journal.pone.0326154

**Published:** 2025-06-27

**Authors:** Miray Unlu Yazici, J. S. Marron, Burcu Bakir-Gungor, Fei Zou, Malik Yousef

**Affiliations:** 1 Department of Bioengineering, Abdullah Gül University, Kayseri, Turkey; 2 Department of Statistics and Operations Research, University of North Carolina, Chapel Hill, North Carolina, United States of America; 3 Department of Computer Engineering, Abdullah Gul University, Kayseri, Turkey; 4 Department of Biostatistics, University of North Carolina at Chapel Hill, Chapel Hill, North Carolina, United States of America; 5 Department of Genetics, University of North Carolina at Chapel Hill, Chapel Hill, North Carolina, United States of America; 6 Department of Information Systems, Zefat Academic College, Zefat, Israel; 7 Galilee Digital Health Research Center, Zefat Academic College, Zefat, Israel; Institute for Basic ScienceREPUBLIC OF KOREA

## Abstract

Breast Cancer (BRCA) is a heterogeneous disease, and it is one of the most prevalent cancer types among women. Developing effective treatment strategies that address diverse types of BRCA is crucial. Notably, among different BRCA molecular sub-types, Hormone Receptor negative (HR-) BRCA cases, especially Basal-like BRCA sub-types, lack estrogen and progesterone hormone receptors and they exhibit a higher tumor growth rate compared to HR+ cases. Improving survival time and predicting prognosis for distinct molecular profiles is substantial. In this study, we propose a novel approach called 3-**M**ulti-**O**mics **N**etwork and Integration **T**ool (3Mont), which integrates various -omics data by applying a grouping function, detecting pro-groups, and assigning scores to each pro-group using Feature importance scoring (FIS) component. Following that, machine learning (ML) models are constructed based on the prominent pro-groups, which enable the extraction of promising biomarkers for distinguishing BRCA sub-types. Our tool allows users to analyze the collective behavior of features in each pro-group (biological groups) utilizing ML algorithms. In addition, by constructing the pro-groups and equalizing the feature numbers in each pro-group using the FIS component, this process achieves a significant 20% speedup over the 3Mint tool. Contrary to conventional methods, 3Mont generates networks that illustrate the interplay of the prominent biomarkers of different -omics data. Accordingly, exploring the concerted actions of features in pro-groups facilitates understanding the dynamics of the biomarkers within the generated networks and developing effective strategies for better cancer sub-type stratification. The 3Mont tool, along with all supporting materials, can be found at https://github.com/malikyousef/3Mont.git.

## Introduction

Cancer as one of the leading causes of death, poses a threat with its incidence rising across the globe. According to American Cancer Society statistics [1], in 2024, breast cancer patients constituted 32% of all new cancer cases among women while prostate cancer accounts for 29% of new cases among men. The annual rise in incidence rates of invasive breast cancer in females, currently at 0.6%, shows a concerning upward trend. Preventive measures and targeted treatment strategies are crucial for reducing the fatality risks. A comprehensive understanding of the underlying mechanisms driving these cancer types is imperative for effective interventions.

The multi-omics integrative approaches facilitate the identification of differentially expressed disease-associated bio-signatures, as well as the molecular classification and prognosis estimation of the diseases. Introducing additional -omics layers, such as metabolomics, proteomics, and transcription factors into existing methodologies provides valuable insights into the understanding of the underlying molecular mechanisms of complex diseases. In cancer tissue analysis, integrating complementary knowledge across different layers allows for a more detailed exploration of tumor architecture and the interactions among bio-signatures at various levels [2,3]. However, the flow of biological information through the layers via integrative approaches presents limitations such as missing values, data heterogeneity, high-dimensionality reduction, and data standardization. Various tools adopting integrative approaches have been developed to overcome these obstacles [4–6]. To achieve strong prediction capability and interpretability for the diagnosis and prognosis of diseases, several machine learning algorithms have been utilized in several studies [7,8]. Identifying the trends or signals with these machine learning models enhances classification performance [9–11], refines the complex patterns among the interacting biomarkers [12,13] and predicts the hub features for therapeutic purposes [14,15]. Moreover, approaches leveraging deep learning algorithms [16–18] have shown success in predicting promising biomarkers for prevention and treatment purposes. However, conventional approaches that are designed to gain new biological and biomedical insights often assess biomolecules individually while building ML models. To generate a more holistic picture, it is important to take into account the *collective functionality of biomolecules*. These biomolecules, which operate in tandem, are called *biological groups*. Via focusing on these groups of molecules, one can conduct intricate analyses in the cellular systems and unveil the molecular dynamics in living tissues. In addition to the grouping methodology employed to grasp the collective behavior of sets of molecules, selecting important features can be challenging, considering dimensionality reduction and accounting for heterogeneity among -omics layers.

To assess the most influential features for distinguishing healthy tissues from tumorous ones, different feature ranking algorithms, such as embedded, filter, and wrapper methods are employed [19]. While effectively mapping -omics data from high to relatively lower-dimensional space, it is necessary to identify the irrelevant features, which helps to reduce the running time via optimizing the ML model. To address the obstacles mentioned above, here we present a novel multi-omics integration tool called 3-**M**ulti-**O**mics **N**etwork and Integration **T**ool (3Mont).

The 3Mont method performs grouping and scoring of the groups using statistical analysis and a machine learning model. The 3Mont is a *biological knowledge-based* improvement over 3Mint [20]. Initially, the groups defined in 3Mint are upgraded to *pro-groups* based on the shared gene lists among the groups. In addition, a new Feature Importance Scoring (FIS) component is implemented within the 3Mont workflow to select the most important features in each pro-group. This step eliminates the effect of pro-group size on the pro-group scores while developing a model for BRCA molecular sub-type identification problem. The effectiveness of each pro-group in BRCA molecular sub-type identification is measured using a Random Forest (RF) model built on 3-omics datasets, which is a major improvement over the 3Mint method that uses only gene expression data. Additionally, 3Mont assesses the importance of pro-groups using the Robust Rank Aggregation (RRA) score [21]. Important signatures (contributing features) and the pro-groups’ feature type distributions (miRNA, methylated CpG sites or mRNA) are provided as summary statistics.

## Materials and methods

### Datasets and preprocessing

The Cancer Genome Atlas (TCGA) Breast Invasive Carcinoma (BRCA) datasets including microRNA (miRNA), gene expression (mRNA), and methylation, were obtained from Xena Public Data Hubs [22]. The tumor samples were classified based on their molecular sub-types as follows: Hormone Receptor positive (HR+) is defined as Estrogen Receptor positive (ER+) and/or Progesterone Receptor positive (PR+). Hormone Receptor negative (HR-) is defined as Estrogen Receptor negative (ER-) and Progesterone Receptor negative (PR-) [23]. 425 samples of HR+ and 124 samples of HR- excluding normal-like sub-types were used to develop a machine-learning model to classify the BRCA molecular subtypes. To prevent class imbalance while creating the ML model, we applied downsampling. In other words, we reduced the number of samples from the majority class to match the number of samples within the minority class. Following a 1:1 ratio, the samples were randomly distributed into the training (90%) and testing sets (10%) in each iteration of the cross-validation step. The preprocessing steps and statistical analyses described in [20] were followed accordingly.

### Proposed 3Mont method

This section provides a comprehensive explanation of the developed approach utilizing preprocessed BRCA datasets. The main objective of the 3Mont is to group the features and arrange the groups based on the shared gene lists among them. The upgraded groups, called pro-groups, have unique names consisting of miRNA/s and CpG/s (methylated CpG sites). Each pro-group consists of one or a few groups. Before assigning scores to each pro-group, the size of the pro-groups is normalized by selecting a fixed number of their features. For this process, the feature sets of the pro-groups are given as input into the FIS (feature importance scoring) component. The most significant features, also called as contributing features, are filtered by a predetermined cut-off value (Gini importance score), selected for each pro-group, and used as input for machine learning model development.

3Mont is based on the notion of decision trees which consist of a set of nodes that recursively split the observations into subsets. The effectiveness of this classification is measured for each node using the Gini impurity. A feature or attribute with the smallest Gini impurity is selected as the split. In this way, the contribution of each feature in a tree is calculated by the decrease in impurity (information gain) and is referred to as the feature importance score (FIS) The sum of the FIS for each feature is averaged over all trees. The smaller the mean FIS value, the less contribution the feature has to the RF model.

**Fig 1:**
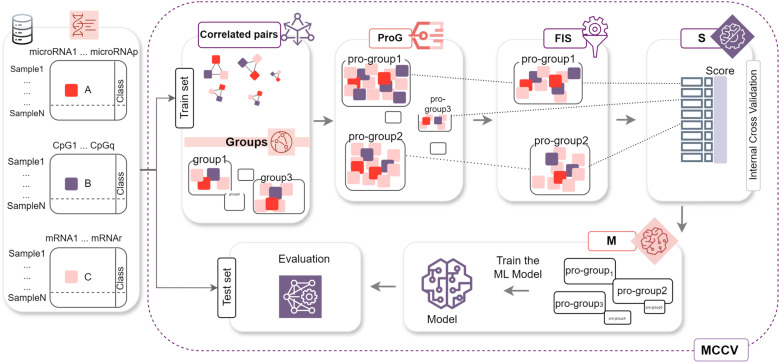
Workflow of 3Mont. The 3Mont pipeline contains four main components: ProG (pro-grouping), FIS (feature importance scoring), S (pro-group scoring), and M (model creation). The features are grouped and groups are arranged based on the shared gene lists among them. Following the normalization of the pro-group sizes using the FIS component, the pro-groups are ordered according to their S component scores. The highest scoring pro-groups are selected to build ML models for classifying BRCA molecular subtypes.

Following that, the pro-group size is determined by sorting the features according to the FIS score and keeping the most important ones, with a pre-determined feature set size, and discarding the rest. In the current study, the pre-determined feature set size is selected as 10, but using FIS, it can be easily modified to select top k features within a pro-group. The 3Mont pipeline is visually depicted in Fig 1.

**Fig 2:**
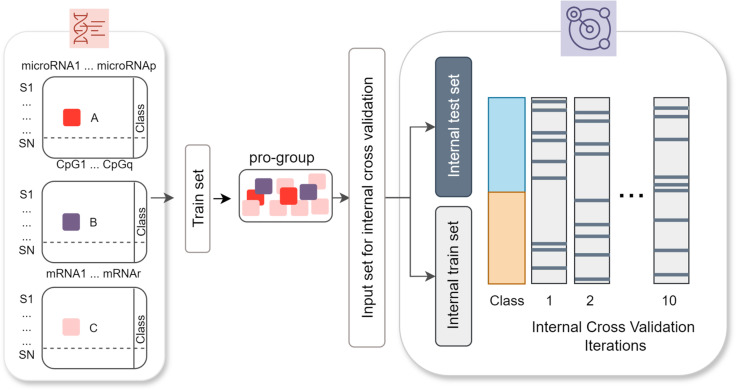
Internal cross-validation step of S component in 3Mont. The expression profiles of each feature and class labels in the ProGroups (represented as a two-class dataset, aggregated from 3 omics datasets) are given as input to the S component. Each dataset is further split into internal training (90%) and internal testing (10%) datasets (encoded by shades of gray). Random splits are repeated 10 times, and the mean accuracy is assigned as the score for each ProGroup.

The next component S is based on scoring each pro-group by applying the RF [24] classifier on the reduced expression datasets via just including the selected (contributing) features within internal cross-validation (shown in Fig 2). The RF model utilizes a random collection of individual decision trees which are combined using a voting schema to obtain more accurate classification [24]. Here, the training set is split into internal training and testing sets for the corresponding features of the pro-group and it is given as an input into the classifier. To this end, the expression values of these features were retrieved from the original -omics datasets. Using these matrices, internal training and test sets are constructed. Then, a score based on the bagging (bootstrap and aggregating) ensemble algorithm is assigned to each pro-group. This step is repeated using shuffle splits in internal cross-validation for each pro-group to prevent selection bias and over-fitting problems in the S component.

Next, the top 10 best-scoring pro-groups are introduced into the following M component as the training data and a RF classifier is developed. The performance of the model is tested with a separate data set to prevent over-fitting. With the use of informative pro-groups, this study aims to distinguish the HR- cases with poor prognostic characteristics [25] from the HR+ ones. Furthermore, the detection of contributing features and the interactions among these features may lead to the identification of promising biomarkers for diseases with heterogeneous characteristics.

To enhance sub-type identification, we have also integrated a community detection-based network analysis as follows. Each pro-group identified by 3Mont consists of miRNA- CpG pairs and their associated features include miRNA(s), CpG ID(s), and/or mRNA(s). Firstly, a network is constructed where each associated feature within the top 10 pro-groups is represented as a node. An edge is formed between any two associated features that co-occur in the same pro-group. Average scores calculated by 3Mont define node weights of associated features. In the current research effort, we focus on the top 10 pro-groups to create a network, but this threshold can be adjusted to include any number of pro-groups. Similarly, in the current study, node size reflects the frequency of each feature in the pro-groups over iterations. However, other parameters can also be utilized to enrich the visualization of the graph.

A community detection algorithm was applied in the generated network and highly interconnected features were identified using the cluster_fast_greedy function from the igraph package [26]. The algorithm iteratively merges the features to increase the modularity score. The maximization of the modularity is evaluated with greedy optimization. In this way, the clusters of related features (communities) were identified.

## Results and discussion

### Size distribution of the identified pro-groups

**Fig 3:**
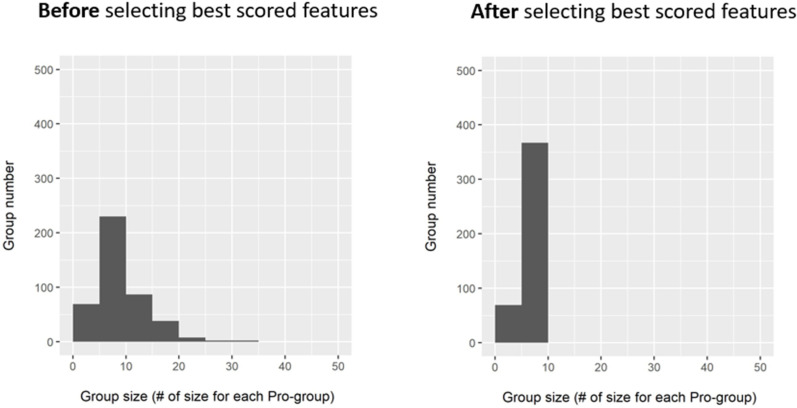
Distribution of pro-group sizes before and after selecting the top 10 features. The size indicates the total number of features within a pro-group. The Gini importance function is used to rank features and the best features in each pro-group are chosen based on their Feature Importance Scores (FIS). In the right panel, a predetermined cut-off value of 10 is used.

Firstly, we evaluate the size distribution of the identified pro-groups for BRCA molecular sub-type identification problem. Here the size refers to the number of features within a pro-group. The pro-group size distributions before and after applying the Gini importance are presented in Fig 3. In the left panel, the sizes of pro-groups range from 1 to 35 before applying the filtering process. After applying the Gini importance function, features with FIS are returned in each pro-group. Based on their FIS, the top 10 features from each pro-group are retained. The right panel shows the size of pro-groups which range from 1 to 10, after applying the filtering process. For training the Random Forest classifier, we decided to keep only the selected top 10 features for each pro-group.

### Performance evaluation of 3Mont

As shown in Fig 1, after selecting the top 10 features within the pro-groups based on their FIS, in the scoring component, the performance of each identified pro-group is assessed using the RF classifier. 10 best-scoring pro-groups are given to the M component to create a model for distinguishing HR+ cases from HR- cases. The 3Mont tool is also tested across multiple cancer types, such as bladder, kidney, liver, and breast cancer subtypes. Table 1 summarizes the performance metrics calculated across these different cancer types. As shown in Table 1, 3Mont generated over 0.85 AUC and over 0.80 accuracy values for all tested cancer datasets.

**Table 1:** Performance evaluation metrics of 3Mont across different cancer types. The total sample size column lists the numbers of the tumor and control samples, respectively. A 1:1 sampling ratio is applied in each iteration of the cross-validation step to prevent class imbalance.

Cancer types	Sample size Case (Control)	Average Feature # for each pro-group	Accuracy	Specificity	Sensitivity	AUC	Precision	Recall	F-measure
**BLCA (tumor vs control)**	408 (17)	8.2	0.80	0.70	0.90	0.85	0.78	0.90	0.83
**BRCA (tumor vs control)**	768 (80)	8.5	0.96	0.94	0.98	0.97	0.95	0.98	0.96
**KIRC (tumor vs control)**	320 (24)	8.0	1.0	1.0	1.0	1.0	1.0	1.0	1.0
**LIHC (tumor vs control)**	367 (44)	8.2	0.91	0.90	0.92	0.96	0.93	0.92	0.92
**PRAD (tumor vs control)**	494 (35)	8.8	0.94	0.92	0.95	0.98	0.94	0.95	0.94
**BRCA (Her2 vs Basal like)**	36 (88)	6.2	0.94	0.92	0.95	0.99	0.94	0.95	0.94
**BRCA (LumA vs LumB)**	302 (123)	9.6	0.82	0.85	0.78	0.87	0.87	0.78	0.82
**BRCA (LumA vs LumB and Her2 and Basal like)**	302 (247)	10	0.81	0.76	0.85	0.89	0.78	0.85	0.81
**BRCA (LumA, LumB vs Her2 and Basal like)**	425 (124)	9.8	0.92	0.91	0.93	0.97	0.92	0.93	0.92

Abbreviations: BLCA: Bladder Cancer, BRCA: Breast Cancer, KIRC: Kidney Renal Clear Cell Carcinoma, LIHC: Liver Cancer (Hepatocellular Carcinoma), PRAD: Prostate Cancer.

The proposed tool facilitates the identification of patterns and correlations of different diseases via identifying molecular signatures. The average number of features denotes the mean feature number of the highest-scoring group in each iteration. For example, 6 features on average are found in the highest scoring group that is identified for the Her2 vs. Basal-like subtypes of BRCA data set, while around 8 features are found for the LIHC data set.

### Significant pro-groups highlighted by 3Mont for BRCA molecular subtype identification (HR+ and HR-)

The top 10 most significant pro-groups and their summary statistics calculated by 3Mont are given in Table 2.

**Table 2:** The 3Mont summary statistics for the top 10 most significant pro-groups that are identified for BRCA molecular subtypes (HR+ and HR-) dataset. These statistics include Frequency of group (the number of times the pro-group appears), Average Score (the score assigned in the S component), Robust Rank Aggregation (RRA) score over 10 iterations. The genes regulated by the methylated CpG site and the pro-group-associated features across -omics datasets are listed in the last two columns, respectively.

Pro-group	Frequency	Average Score	RRA Score	Avg Rank	Associated Features
**hsa-mir-135b_cg14621217**	4	0.93	0.0005	1.75	GATA3(1), MLPH(4), PPP1R14C(3), SLC44A4(2), AGR2(4), FOXA1(4), UGT8(1), SPDEF(4), FOXC1(3), AR(2), FBP1(1), SFT2D2(2), C9ORF152(1), PRR15(2), SIDT1(1), hsa-mir-135b(2), SRSF12(1), cg14621217(1), LINC02188(1)
**hsa-mir-135b_cg08993690**	6	0.91	0.0001	2.5	PPP1R14C(6), MLPH(6), AGR2(3), FOXA1(6), AR(2), SLC44A4(2), SFT2D2(2), VGLL1(2), FOXC1(6), SPDEF(4), PRR15(3), SIDT1(3), cg08993690(4), UGT8(2), LINC02188(3), FBP1(1), C9ORF152(1), hsa-mir-135b(3), STAC(1)
**hsa-mir-135b_cg07810039**	1	0.94	0.011	2	FOXA1(1), SLC44A4(1), FBP1(1), MLPH(1), AGR2(1), cg07810039(1), PPP1R14C(1), SPDEF(1), SFT2D2(1), C9ORF152(1)
**hsa-mir-135b_cg12739419**	1	0.92	0.012	2	MLPH(1), PRR15(1), FOXA1(1), AGR2(1), SPDEF(1), PPP1R14C(1), cg12739419(1), FOXC1(1), hsa-mir-135b(1), SIDT1(1)
**hsa-mir-135b_cg10558233, hsa-mir-135b_cg23093870**	1	0.96	0.019	1	GATA3(1), PPP1R14C(1), FOXA1(1), MLPH(1), AGR2(1), SLC44A4(1), cg10558233(1), SFT2D2(1), AR(1), VGLL1(1)
**hsa-mir-577_cg07212543, hsa-mir-577_cg08452338, hsa-mir-577_cg08993690, hsa-mir-577_cg12427162, hsa-mir-577_cg13975098, hsa-mir-577_cg14652095, hsa-mir-577_cg17806482, hsa-mir-577_cg22161115, hsa-mir-577_cg23093870, hsa-mir-577_cg24051242, hsa-mir-577_cg24914185, hsa-mir-577_cg25309292, hsa-mir-577_cg25883066, hsa-mir-577_cg25979244**	1	0.897777778	0.023809524	3	cg24914185(1), MLPH(1), AGR2(1), FOXA1(1), cg17806482(1), cg24051242(1), cg22161115(1), cg25979244(1), PRR15(1), cg13975098(1)
**hsa-mir-135b_cg07810039, hsa-mir-135b_cg09182138**	1	0.91	0.02	2	MLPH(1), AGR2(1), FOXA1(1), PRR15(1), cg07810039(1), SPDEF(1), PPP1R14C(1), SIDT1(1), AR(1), FOXC1(1)
**hsa-mir-577_cg02212575, hsa-mir-577_cg07185041, hsa-mir-577_cg09246948, hsa-mir-577_cg14986386, hsa-mir-577_cg17041511, hsa-mir-577_cg24296761**	1	0.911111111	0.026	2	MLPH(1), cg02212575(1), FOXA1(1), cg24296761(1), AGR2(1), cg14986386(1), PRR15(1), SFT2D2(1), cg07185041(1), FOXC1(1)
**hsa-mir-577_cg11524039**	9	0.896296296	0.027	2.7	MLPH(8), cg11524039(8), AGR2(7), FOXA1(8), SLC44A4(2), FOXC1(4), SIDT1(8), UGT8(8), hsa-mir-577(8), SOX9-AS1(1), PRR15(5)
**hsa-mir-577_cg06411879, hsa-mir-577_cg09077126, hsa-mir-577_cg11524039, hsa-mir-577_cg12427162, hsa-mir-577_cg14652095, hsa-mir-577_cg17580935, hsa-mir-577_cg23093870, hsa-mir-577_cg24051242, hsa-mir-577_cg25883066, hsa-mir-577_cg25979244**	1	0.917777778	0.03	2	AGR2(1), FOXA1(1), MLPH(1), cg11524039(1), SLC44A4(1), cg12427162(1), cg24051242(1), PRR15(1), cg09077126(1), cg25979244(1)

In Table 2, a lower rank indicates stronger statistical significance. The frequency column indicates the number of times the pro-group appears (out of 10 iterations). The average score which is the output of the S component, indicates the mean of the score of the pro-group over the iterations. The Robust Rank Aggregation (RRA) score [21] tracks the rank of each feature over the iterations, giving a score in the range 0–1. Lower RRA scores indicate stronger significance of the pro-groups. The average rank is the mean of the ranks assigned to each pro-group in each iteration. The RRA scores and average ranks in Table 1 have some correlation with each other but convey different useful information.

Based on the RRA scores, the top 10 pro-groups identified for the BRCA molecular sub-type data set are given in Table 2. This table indicates that the miRNA hsa-miR-135b is involved in the discrimination of the HR + /HR- classes for BRCA. It is reported in [27] that the miRNA-135b is a diagnostic biomarker for Triple Negative Breast Cancer (TNBC), including basal-like subtypes, compared to non-TNBCs. Upregulation of miRNA-135b induces the migration process and tumorigenesis by affecting WNT and Hippo signaling regulators [28,29]. Some of those miRNAs, CpGs, and target genes, which are identified by 3Mont for the BRCA molecular sub-type data set have been reported in the literature as follows. Fan *et al.* showed that hsa-miR-577 has prognostic relevance for TNBC molecular heterogeneity compared to non-TNBC phenotypes [30], where this miRNA is also detected in our analysis.

At the methylation level, the differentially methylated gene *SFT2D2* contributes to the metastatic behavior of BRCA cases [31]. A methylation level change in the gene *TBCD*, which has role in Post-chaperonin tubulin folding pathway [32], has been identified with 3Mont. BRCA patients with induced expression of the *SERTAD2* gene, also known as *TRIP-Br-2*, showed a positive association with tumorigenesis. *TRIP-Brs* contribute to central carbon metabolism [33]. However, the association of these potential biomarkers has not yet been clearly identified with BRCA subtypes.

### Relations between the detected biomarkers for BRCA molecular subtype (HR + /HR- cases) identification

**Fig 4:**
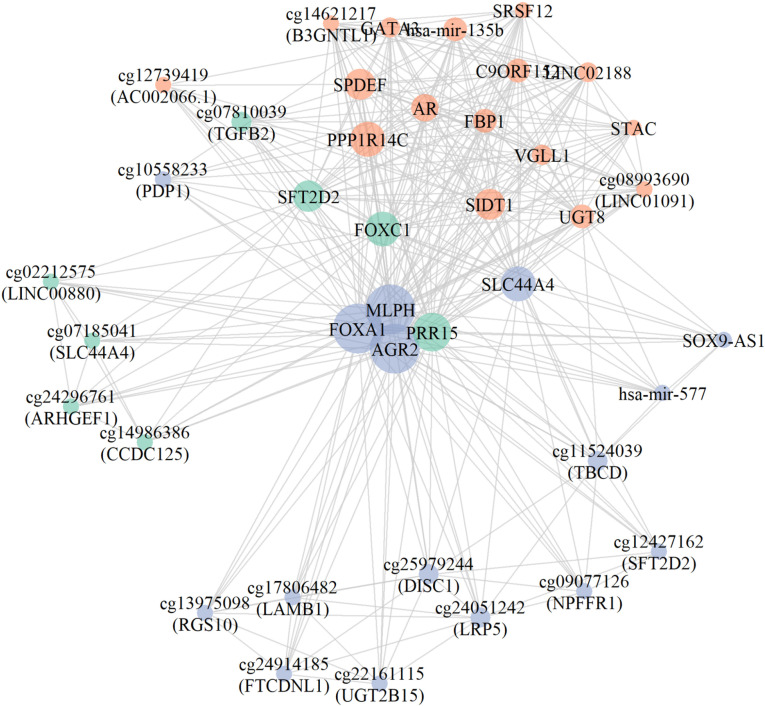
Generated network illustrating the pro-groups and their associated features that are identified by 3Mont for differentiating among the BRCA molecular subtypes (HR + /HR- cases). The network visualizes associations between mRNAs, CpG IDs, miRNAs within the pro-groups. The node size represents the scaled average score of each associated feature within the top 10 pro-groups. The scores are obtained from the Average scores column of Table 2. Different colors represent distinct communities (clusters) detected by the community detection algorithm.

To understand the relations among the identified biomarkers, we followed a network analysis as explained in the methods section. Fig 4 presents the generated network based on the pro-groups and their associated features identified by 3Mont when applied to the BRCA molecular sub-type data set (HR + /HR- cases). Fast greedy modularity maximization algorithm is used to generate the graph, where the node size represents the average score (obtained from Table 2). The network structure is formed by utilizing the connections obtained from 3Mont (features in the same pro-groups are connected). This is the primary driver for the community detection in this analysis. Nodes represent genes (mRNAs), miRNAs and CpG sites (methylation regions) within the top 10 pro-groups. Edges between the nodes imply that these associated features exist within the same pro-group. Three different colors represent the three distinct communities identified within this network.

Several nodes in Fig 4 including *AGR2, FOXA1, FOXC1, GATA3, MLPH, PPP1R14C, SFT2D2, SPDEF, SRSF12, UGT8, VGLL1,* cg14621217 (*B3GNTL1*) and cg12427162 (*SFT2D2*), have been reported to be associated with TNBC (Triple Negative Breast Cancer) [31]. These nodes refer to the associated features that are identified by 3Mont for the top 10 pro-groups. It was shown that *POLR3G* expression is negatively correlated with *FOXA1* and *AR* expression in TNBC [34]. Additionally, the potential therapeutic targets C9orf152 and *UGT8* were reported in the onset of TNBC cases [35,36]. The blue community in Fig 4 plays a central role with its key nodes (*AGR2, FOXA1, MLPH*) and it serves as a bridge for other communities. These key nodes’ connections with *PRR15* and *FOXC1* in the green community suggest a functional interplay to distinguish the HR + /HR- classes.

One of the most frequently appearing genes in our analysis, *FOXA1,* has been reported in [37] to induce ER+ BRCA endocrine resistance. It has been reported that *FOXC1,* another commonly detected gene in 3Mont analysis, induces Hedgehog signaling and serves as a potential prognostic marker in Basal-like BRCA sub-type, displaying aggressive behavior and poor prognosis [38]. Furthermore, the gene *GATA3,* represented in both Estrogen-dependent gene expression and ESR-mediated signaling pathways, is mutated with high frequency in Luminal subtypes of BRCA [39]. The collaborative action of the genes *GATA3*, *ER-α*, and *FOXA1* contributes to the differentiation status of Luminal vs Basal-like BRCA cases [40,41]. The over-expression of the gene *AGR2* and its interacting biomarker *FOXA1* have been shown to decrease the prognosis of ER+ BRCA cases [42].

Furthermore, functional enrichment analysis using Gene Ontology terms is performed to reveal distinct biological processes for the identified features shown in Fig 4 and Table 2. The genes *AR, FOXA1* and *TGFB2* play role in gland, epithelial tube morphogenesis and positive regulation of cell differentiation [43,44], while *GATA3* is involved in mammary gland duct morphogenesis and uterus development [45]. *AGR2* and *SPDEF* contribute to the negative regulation of epithelial to mesenchymal transition [46,47]. The CpG site cg24051242 (*LRP5*) has role in mammary gland duct morphogenesis and epithelium development [48], while cg17806482 (*LAMB1*) and cg25979244 (*DISC1*) are involved in positive regulation of cell differentiation and positive regulation of developmental process [49,50] in cancer tissue. The CpG site cg14986386 (*CCDC125*) has roles in negative regulation of Ras protein signal transduction and positive regulation of molecular function [51]. Another CpG site cg13975098 (*RGS10*) is involved in molecular function and negative regulation of signal transduction [52]. The involvement of these features in these biological processes suggests their potential contribution to tumor metastasis and disease progression mechanisms.

### Comparative performance evaluation of 3Mont

**Fig 5:**
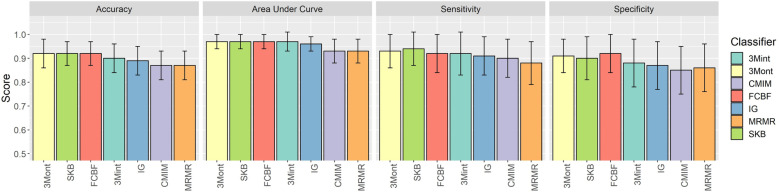
Comparative performance evaluation of 3Mont with other feature selection algorithms over 10 iterations. The average performance metrics of the best scoring groups, along with standard deviations over iterations, are shown in panels which are labeled as accuracy, Area Under ROC Curve, sensitivity and specificity. All algorithms use mRNA, miRNA, and methylation data, but 3Mint only uses mRNA expression in training and testing the classifier. Abbreviations: SKB: SelectKBest, FCBF: Fast Correlation Based Filter, IG: Information Gain, CMIM: Conditional Mutual Information Maximization, MRMR: Minimum Redundancy Maximum Relevance.

Fig 5 compares the performance metrics of 3Mont, 3Mint, and some other feature selection algorithms. All algorithms are run using all feature types (mRNA expression, miRNA expression and methylation) except 3Mint which uses only mRNA expression profiles. While the number of features is defined by the highest scoring group in 3Mint (15 genes), other algorithms perform the analysis with the feature number filtered to 10. In 3Mont, after selecting the best-scoring features within each pro-group, the S component is applied to assign a score to each pro-group. Subsequently, the 10 highest-scoring pro-groups are used to train and test the RF classifier. While differentiating between the HR + vs. HR- cases, 3Mont, SKB (Select K Best) and FCBF (Fast Correlation Based Filter) feature selection methods have the highest classification performance. Among the 3 best-performing feature selection methods, 3Mont and SKB share the features of *GATA3*, cg23205034 targeting the *CPQ* gene, cg10970143 targeting *CFAP45* gene, hsa-mir-934; while SKB and FCBF have hsa-mir-4766, hsa-mir-3682, *ZNF454* in their common features list. There are no shared features between 3Mont and FCBF.

## Discussion

There are various strengths of 3Mont over traditional feature selection algorithms. Our tool integrates multiple functions through the development of ProG (pro-grouping), FIS (feature importance scoring), S (pro-group scoring), and M (model creation) components. Initially, 3Mont selects correlated features based on their expression profiles obtained from -omics datasets. Then our tool groups the features if they have common gene(s). Defining groups (each one having unique miRNA and CpG site names) with associated gene(s) distinguishes our tool and 3Mint from other feature selection methods.

Machine learning algorithms typically select features one by one and ignore grouping strategies and the identification of correlated features using statistical approaches. However, 3Mont’s novel ProG component provides a collection of groups with common gene(s) and prevents the repetition of features within groups. Another aim of 3Mont is to select the hub features in each group and identify the significant features for sub-type differentiation. To achieve this, the FIS component is applied to filter the features in each group. These features, called contributing features, are then used to develop machine learning models.

By integrating statistical and ML methods with a grouping approach, 3Mont offers a more comprehensive tool for determining the collective behaviour of features compared to traditional feature selection algorithms. Additionally, restricting the number of features in groups via the FIS component decreases the processing time for sub-type classification in 3Mont compared to 3Mint. Feature interaction networks are constructed in 3Mont to illustrate the interplay of significant biomarkers.

## Conclusion

Integration of multi-omics data has enhanced our understanding of heterogeneous diseases, providing a holistic view at the molecular level. The method offered in this study provides opportunities to identify novel bio-signatures, and personalized treatment approaches. 3Mont method categorizes biologically important groups of features across various -omics datasets into biological groups. This approach enables researchers to gain novel insights into the collective functionality among biomolecules. The new concept of pro-groups extends previously defined biological groups with the inclusion of shared features. The construction of pro-groups reduces the number of groups through merging operations. Because pro-groups with more features tend to have higher scores, the sizes of pro-groups are equalized to eliminate the effect of pro-group size in the scoring component. These equalizing pro-groups are utilized for developing an ML model to tackle the classification problem. Finally, the feature importance scoring component enables us to pinpoint the most impactful bio-signatures in each pro-group.

With our new diagnostic models developed in this study, we identified promising biomarkers such as miRNA-135b, *GATA, FOXA1,* and differentially methylated gene *DISC1* to distinguish the BRCA subtypes (HR + /HR- cases). Although 3Mont is used to identify BRCA molecular subtypes in the current study, this approach can also be applied to predict disease progression based on the class labels provided in the data set. The future strategies for 3Mont include the incorporation of additional -omics datasets such as transcription factors and metabolomics data to provide deeper insights into the cellular mechanisms underlying diseases. Another direction is the optimization of feature selection and grouping approaches for 3Mont by utilizing advanced statistical techniques to improve the efficiency and performance of the tool. Expanding the tool to analyze various disease datasets will enable novel bio-signature discovery and accelerate the development of therapeutic approaches.

## Supporting information

S1 File Evaluation of the effect of downsampling with descriptive analysis. (DOCX)

S2 File Comparative Evaluation using the BRCA molecular subtype dataset. (DOCX)

S3 File Tissue-specific gene enrichment analysis of the identified biomarkers. (DOCX)
