# Supplementary material for: 3Mont: A multi-omics integrative tool for breast cancer subtype stratification
Source: PLoS One. 2025 Jun 27;20(6):e0326154. doi: 10.1371/journal.pone.0326154 (PMC12204537; doi:10.1371/journal.pone.0326154)
Supplement: S1 File — (DOCX) [file pone.0326154.s001.docx]

# **Supporting information**

*Evaluation of the effect of downsampling:* To assess the impact of downsampling, we performed a descriptive analysis of the features within the top-scoring pro-groups before and after filtration. S1 Fig illustrates the normalized expression values (shown in y-axis) and class information (shown in x-axis). The color key refers to pre- and post-filtration stages. Each subgraph shows a feature (identified biomarker) from the top-ranked pro-groups. Our findings imply that there are no significant differences before and after filtration, supporting the validity of our machine learning approach with downsampling. The descriptive statistics and feature importance remain stable, reinforcing the robustness of our methodology.

**
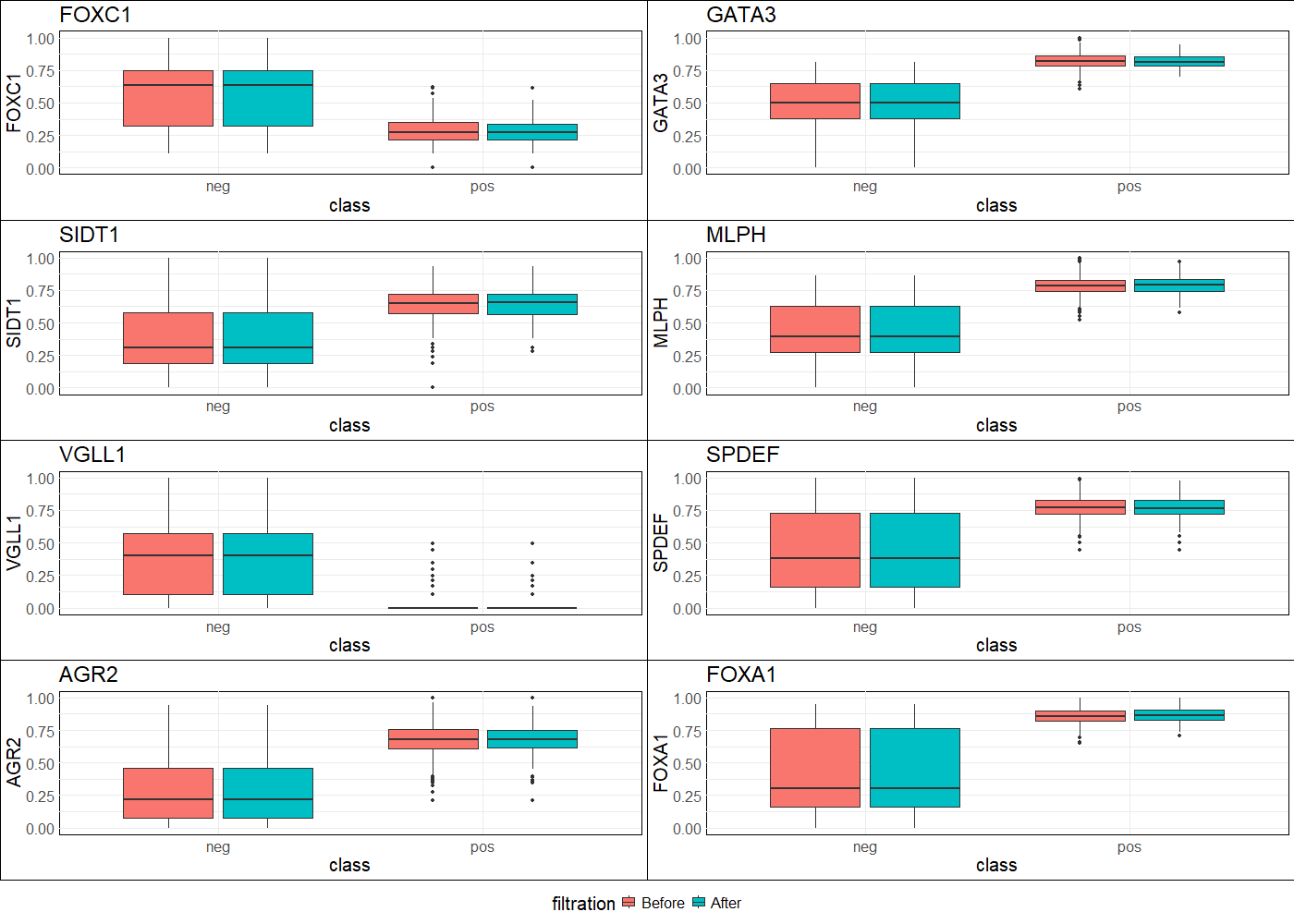
**
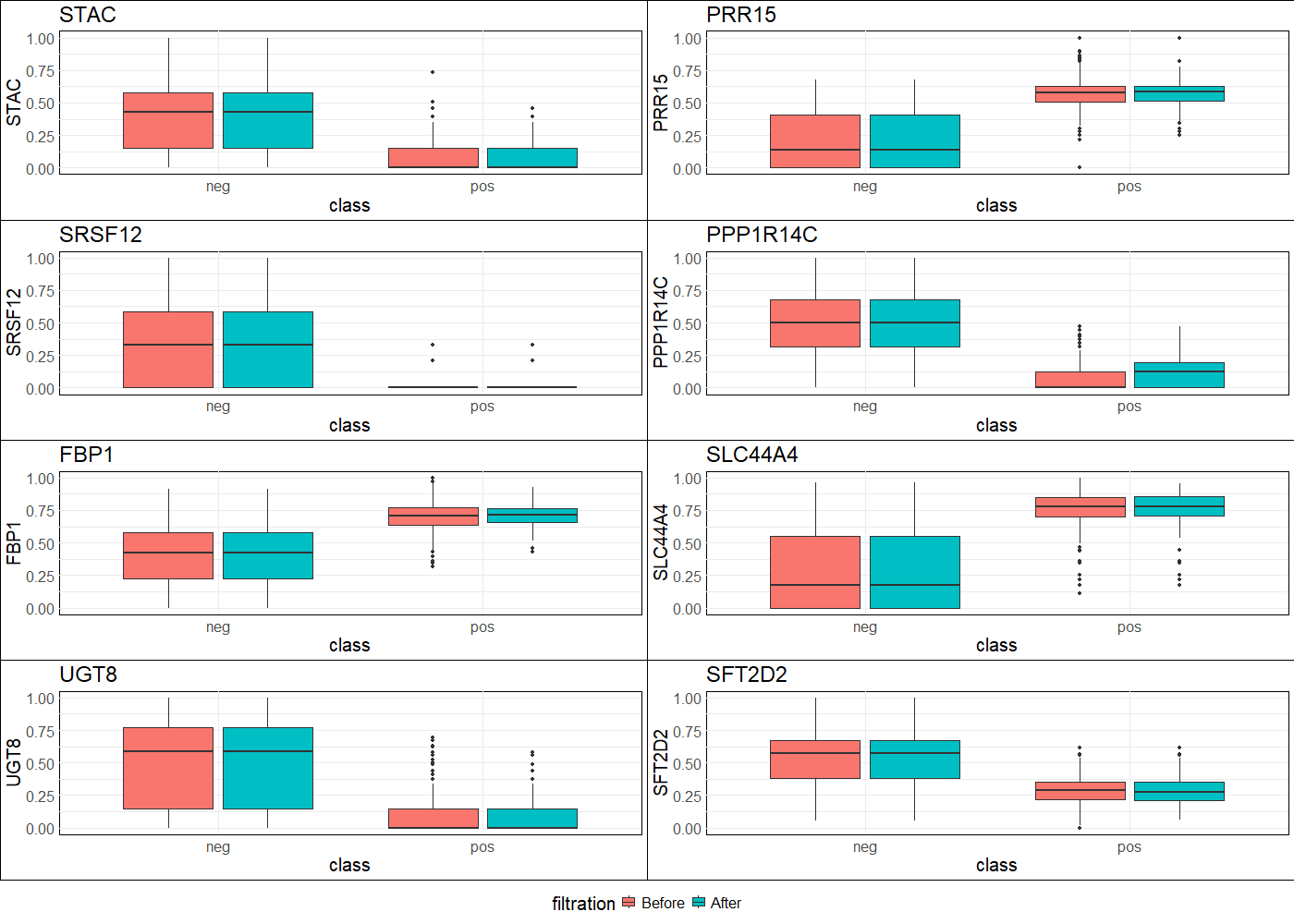


**S1 Fig. Descriptive analysis of the features within the top-scoring pro-groups before and after filtration (downsampling). Normalized gene expression values of identified biomarkers before and after downsampling, are shown in red and green, respectively. The BRCA molecular subtype dataset is used in this descriptive analysis, where ‘neg’ denotes HR+ and ‘pos’ denotes HR- Breast cancer classes.**
