# Supplementary material for: 3Mont: A multi-omics integrative tool for breast cancer subtype stratification
Source: PLoS One. 2025 Jun 27;20(6):e0326154. doi: 10.1371/journal.pone.0326154 (PMC12204537; doi:10.1371/journal.pone.0326154)
Supplement: S2 File — (DOCX) [file pone.0326154.s002.docx]

# **Supporting information**

*Comparative Evaluation:* Using the BRCA molecular subtype dataset, we compare our findings with the findings of mixOmics (Rohart et al, 2017) which also performs m[ulti-omics data integration](https://mixomics.org/). S2 Fig shows that while some of the biomarkers are commonly identified in both methods, some biomarkers are uniquely suggested by our tool and some others are reported only by mixOmics. The genes *AGR2, PRR15, GATA3, MLPH* and *FOXA1*; the miRNAs hsa-mir-135b and hsa-mir-577; the CpG sites cg24051242, cg17806482, cg25979244, cg12427162 and cg24296761 are the common important features among 3Mont and mixOmics.


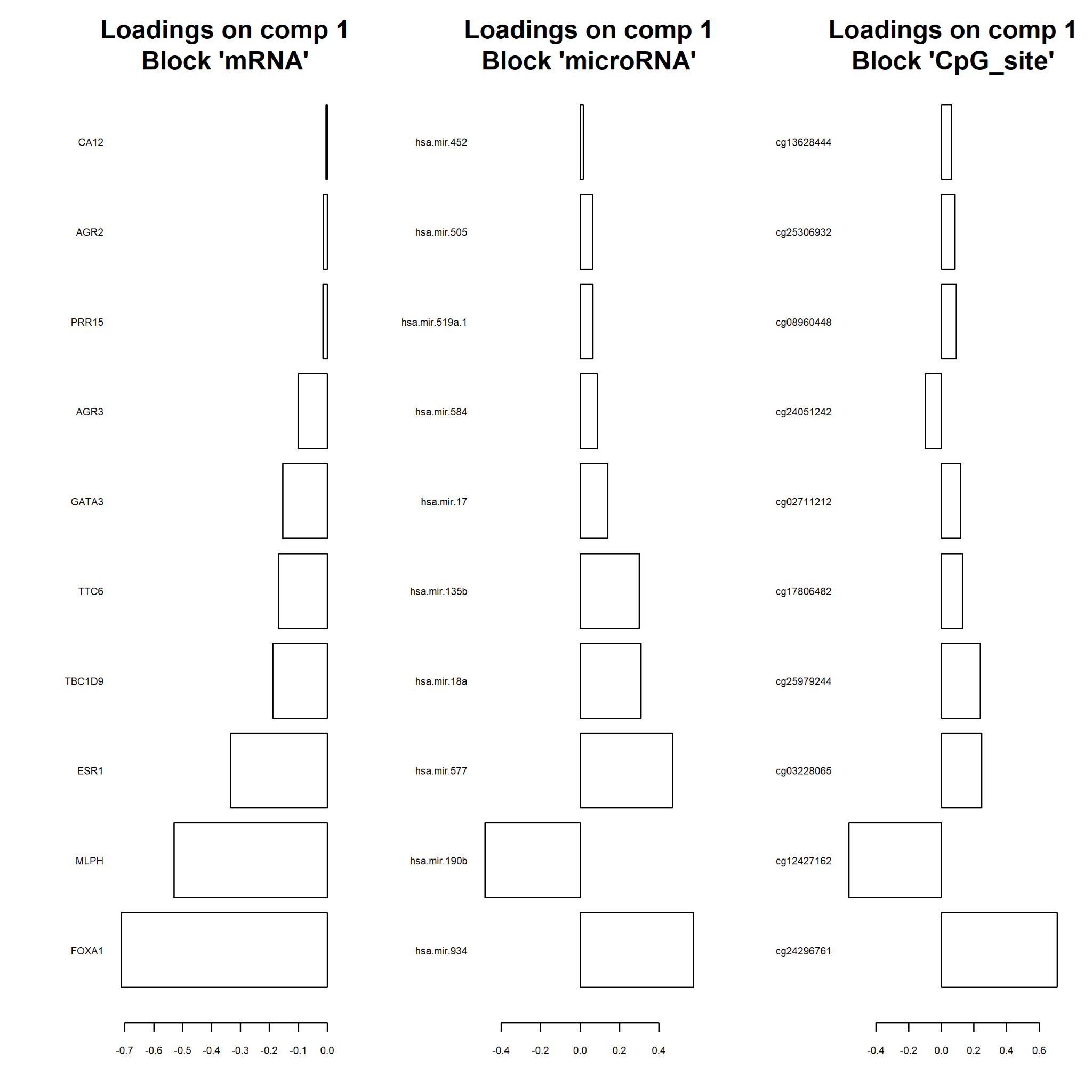


**S2 Fig. The key features identified by mixOmics for differentiating the HR+ and HR- breast cancer subtype cases using 3-omics datasets (mRNA and microRNA expression data and methylated CpG sites). Each block represents the most significant features identified by mixOmics. The strength of each feature’s contribution in discriminating the classes is represented with the horizontal bars. The sign of the loading indicates the positive or negative relationships of the feature on the component in separating the subtypes.**

**References**

Rohart F., Gautier, B, Singh, A and Lê Cao, K. A. mixOmics: an R package for 'omics feature selection and multiple data integration. PLOS 2-17.
