# Supplementary material for: 3Mont: A multi-omics integrative tool for breast cancer subtype stratification
Source: PLoS One. 2025 Jun 27;20(6):e0326154. doi: 10.1371/journal.pone.0326154 (PMC12204537; doi:10.1371/journal.pone.0326154)
Supplement: S3 File — (DOCX) [file pone.0326154.s003.docx]

# **Supporting information**

*Tissue-specific gene enrichment analysis of the identified biomarkers:* Using the biomarkers identified in Table 2 and shown in Fig 4, we perform tissue-specific gene enrichment analysis using STRING. The relevance of these features with the most relevant tissues was highlighted in S3 Fig.


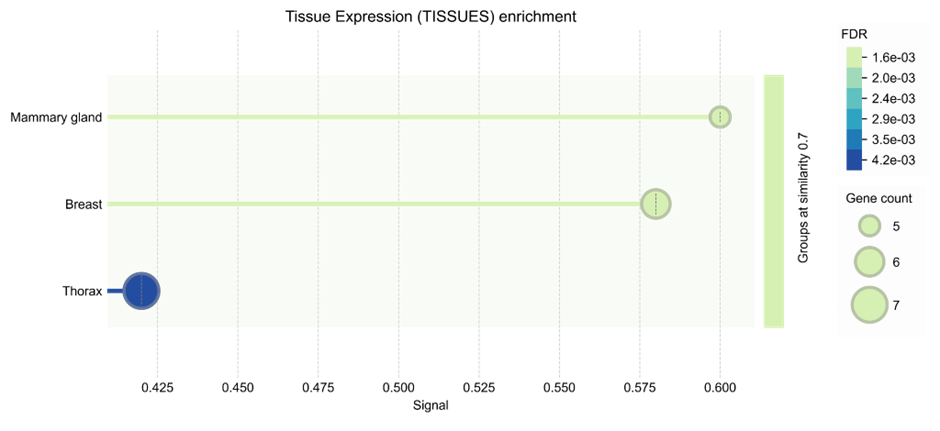


**S3 Fig. Tissue-specific patterns that are identified with tissue expression enrichment analysis for the features detected by 3Mont. While the signal axis represents the enrichment level within each tissue group, groups at similarity score (vertical bar) indicate tissue clustering. The size of each circle shows the number of genes enriched in the corresponding tissue. The color key represents false discovery rate (FDR) where lighter colors indicate higher significance.**

The expression of *FBP1* gene is mostly observed in the thorax while the expression of *MLPH* gene is mostly observed in both breast and thorax tissues. The genes *UGT2B15, SLC44A4, AGR2, SPDEF FOXA1* exhibited expression across several tissues including mammary gland, breast and thorax.
